# mTOR Pathway in Gastroenteropancreatic Neuroendocrine Tumor (GEP-NETs)

**DOI:** 10.3389/fendo.2020.562505

**Published:** 2020-11-16

**Authors:** Sara Zanini, Serena Renzi, Francesco Giovinazzo, Giovanna Bermano

**Affiliations:** ^1^Centre for Obesity Research and Education (CORE), School of Pharmacy and Life Sciences, Robert Gordon University, Aberdeen, United Kingdom; ^2^School of Biosciences and Veterinary Medicine, University of Camerino, Camerino, Italy; ^3^Fondazione Policlinico Universitario A. Gemelli Istituto di ricovero e cura a carattere scientifico (IRCCS), Department of Surgery -Transplantation Service, Rome, Italy

**Keywords:** neuroendocrine tumor, mTOR, cancer treatment, target therapy, GEP-NENs, GEP-NETs

## Abstract

Gastroenteropancreatic neuroendocrine neoplasms (GEP-NENs) originate from neuroendocrine cells in the gastrointestinal tract. They are heterogeneous, and though initially considered rare tumors, the incidence of GEP-NENs has increased in the last few decades. Therapeutic approaches for the metastatic disease include surgery, radiological intervention by chemoembolisation, radiofrequency ablation, biological therapy in addition to somatostatin analogs, and PRRT therapy (177Lu-DOTATATE). The PI3K-AKT-mTOR pathway is essential in the regulation of protein translation, cell growth, and metabolism. Evidence suggests that the mTOR pathway is involved in malignant progression and resistance to treatment through over-activation of several mechanisms. PI3K, one of the main downstream of the Akt-mTOR axis, is mainly involved in the neoplastic process. This pathway is frequently deregulated in human tumors, making it a central target in the development of new anti-cancer treatments. Recent molecular studies identify potential targets within the PI3K/Akt/mTOR pathway in GEP-NENs. However, the use of target therapy has been known to lead to resistance due to several mechanisms such as feedback activation of alternative pathways, inactivation of protein kinases, and deregulation of the downstream mTOR components. Therefore, the specific role of targeted drugs for the management of GEP-NENs is yet to be well-defined. The variable clinical presentation of advanced neuroendocrine tumors is a significant challenge for designing studies. This review aims to highlight the role of the PI3K/Akt/mTOR pathway in the development of neuroendocrine tumors and further specify its potential as a therapeutic target in advanced stages.

## Introduction

Gastroenteropancreatic neuroendocrine neoplasms (GEP-NENs) are defined as a heterogeneous group of neoplasia that originates from neuroendocrine cells widely dispersed throughout the gastrointestinal tract forming the largest group of hormone-producing cells in the body (1, 2). Although initially considered rare tumors, in the last decade, the incidence has significantly increased. Different factors may explain this increase such as a better classification with the introduction in 2010 of the World Health Organization (WHO) criteria and the ever-increasing use of screening and diagnostic methods such as the gastrointestinal endoscopy and radiological techniques (3, 4). On the contrary, during the same period, progress in diagnosis has only been matched by a modest improvement in outcomes due to (5) GEP-NENs often being unpredictable and unusual in terms of symptoms, disease progression, and overall survival (6).

Functioning GEP-NENs release peptides and neuroamines that are implicated in specific clinical syndromes, such as carcinoid syndrome, which is relatively uncommon (10–15%) and non-specific symptoms such as irritable bowel syndrome, asthma, or food allergy response. The consequence of late diagnosis (5–7 years on average) is that 75% of tumors exhibit synchronous liver metastases at the time of diagnosis (7, 8). Moreover, 50% of the tumors are asymptomatic until late presentation with symptoms of mass effects or distant metastases, frequently hepatic, or both or tumor-induced fibrosis (9).

Most GEP-NENs are sporadic with a minor group related to inheritable genetic conditions such as multiple endocrine neoplasia type 1 (MEN1), tuberous sclerosis (TSC) and Von-Hippel Lindau (VHL) syndrome (10). Management treatment includes surgery, which at present, is the only therapeutic option in localized and locally advanced disease. Other therapeutic approaches for the metastatic disease include radiological intervention by chemoembolisation, radiofrequency ablation, biological therapy, somatostatin analogs (SSAs), and Peptide Receptor Radionuclide Therapy (PRRT) with 177Lu-DOTATATE (11). Therefore, the need to develop novel therapeutic approaches is paramount in the absence of several treatment strategies.

Frequently, an mTOR abnormal activation has been observed, likely due to inactivating mutations occurring on genes coding for negative regulators of the pathway or through indirect mechanisms. Clinically, the overexpression of mTOR and the downstream targets has been associated with the worst prognosis in different NETs (12–14). Molecularly targeted drugs are emerging as a new and promising treatment for patients affected by GEP-NENs (15).

This review aims to highlight the role of the PI3K/Akt/mTOR pathway in the development of a neuroendocrine tumor and its potential as a therapeutic target providing a biomolecular overview and reporting results from clinical trials.

## Overview of Akt-mTOR Signaling

The phosphatidylinositol 3-kinase (PI3K)-Akt-mTOR pathway supports the modulation of cell growth, proliferation, metabolism, survival, and angiogenesis (16). Evidence suggests that PI3K, one of the significant upstream of the Akt-mTOR axis, is involved in the neoplastic process through the receptor tyrosine kinases (RTKs) and the G protein-coupled receptors (GPCRs). Oncogenic factors such as epidermal growth factor receptor (EGFR), platelet-derived growth factor receptor (PDGFR), and mesenchymal-epithelial transition factor can activate PI3K by binding RTKs and GPCRs (17–19). PI3K is anchored to the plasmatic membrane through a lipid tail. It transduces the signals into intracellular messages by phosphorylating the 3′-OH position of the inositol ring of the lipid second messenger phosphatidylinositol (4, 5) bisphosphate (PIP_2_). Subsequently, phosphatidylinositol (3–5) triphosphate (PIP_3_) recruits and activates the phosphatidylinositol-dependent kinase 1 (PDK1) that phosphorylates the serine-threonine protein kinase AKT [also known as protein kinase B (PKB)] (20).

**Figure 1:**
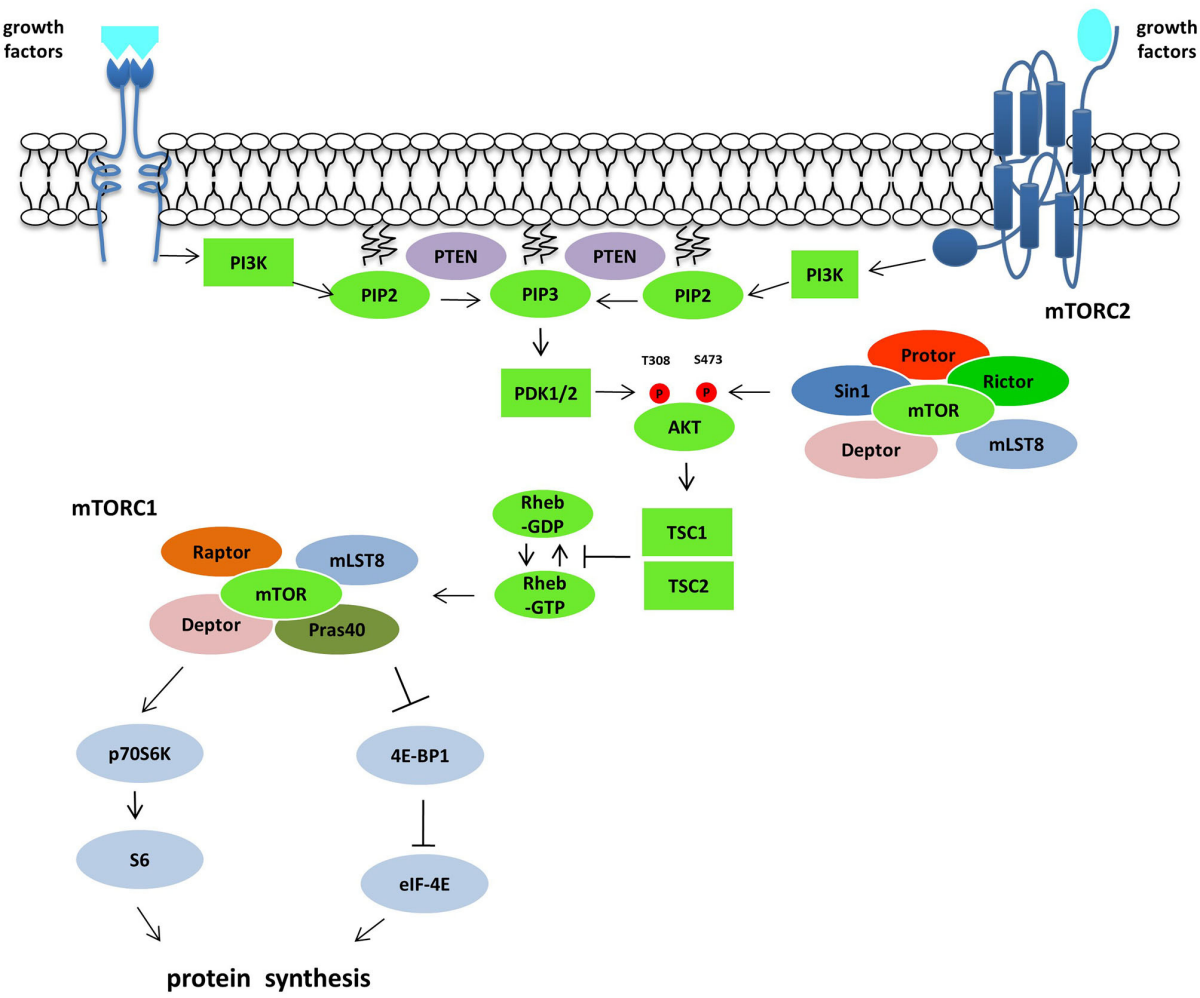
The PI3K/Akt/mTOR pathway. Following growth factors stimulation and subsequent activation of RTKs and GPCRs, PI3K is recruited to the plasma membrane directly or through adaptor protein and phosphorylated PIP2 producing PIP3, which recruits and activates PDK1. Akt activation is mediated by PDK1 on T308 and by mTORC2 complex on S473. Akt controls the activity of mTORC1 inactivating the GTPase activity of the TSC1/TSC2 complex toward the mTORC1 activator Rheb. mTORC1 activation induces protein synthesis *via* p70S6K and 4EBP1. The tumor suppressor gene PTEN acts on this pathway antagonizing the PI3K action on PIP3.

AKTs are serine-threonine kinases and comprise three different protein isoforms (AKT1, AKT2, and AKT3) acting on cellular survival, proliferation, growth, and metabolism. To be fully activated AKT needs the phosphorylation on T308 by PKD1 and S473 by mTORC2. AKT downstream effectors are implicated in the control of apoptosis (FOXO family of transcription factors, BAD or NF-κB), cell cycle regulation (GSK3β, p27kip1), and growth (TSC2) (21). AKT downstream is mTOR that plays a vital role in the regulation of cell growth and proliferation. The control is achieved by controlling cellular energy levels, nutrient availability, oxygen levels, and mitogenic signals. The protein is a serine-threonine protein kinase of the PI3K superfamily, referred to as class IV PI3Ks, frequently overactivated in cancer (22). mTOR is comprised of two complexes, mTOR complex 1 (mTORC1) and mTOR complex 2 (mTORC2), different in chemical structures and substrate specificity. mTORC1 consists of mTOR protein, the regulatory-associated protein of mTOR (raptor), deptor, mlST8, and Pras40 (16). After the stimulation with growth factors as IGF-1 and 2, PDGF and VEGF, the mTORC1 translation is increased via the ribosomal protein S6 kinase (p70S6K) and the eukaryotic initiation factor 4E binding protein (4E-BP1) (23). The function of mTORC1 is modulated within the PI3K/Akt pathway via phosphorylation alongside inactivation of the tuberous sclerosis complex (TSC1/TSC2) by inhibition of the guanosine triphosphatase activity, which controls the activity of the mTOR activator Rheb (22). Tumor suppressor genes, such as phosphatase and tensin homolog (PTEN), that antagonize the PI3K action on PIP_3_ (24), NF1, the kinase LKB1 and oncogenes such as Ras and Raf, all converge on the TSC1/TSC2 complex (25). The activity of HIF1α and VEGF (26) is enhanced through the activation of the mTOR pathway. In contrast, mTORC2 complex is associated with Protor, SIN1, the rapamycin-insensitive companion of TOR protein (Rictor), LST8, Deptor which reacts to growth factor receptor binding, thus initiating full activation of Akt kinase by phosphorylation at the Ser473 (16). This pathway is frequently deregulated in human tumors, making it a central target in the development of new anti-cancer treatments (21) (Figure 1).

## Role of Akt-mTOR Signaling Pathway in GEP-NENs

In the last decade, molecular studies (12, 27, 28) pointed to several targets of the PI3K/Akt/mTOR pathway in GEP-NENs. Shah et al. found that, respectively 76 and 96% of 98 NENs tissues analyzed by IHC display constitutive AKT phosphorylation and activated ERK, a downstream target (29). Missiaglia et al. (30) demonstrated that the expression of two endogenous inhibitors of the mTOR pathway, PTEN and TSC2, were downregulated in a large proportion of tumors, respectively 35 and 60% of cases. Further, low expression was significantly related to both diminished disease-free and overall survival. Overexpression of mTOR has been demonstrated in poorly differentiated NENs, but the expression rate decreased in well-differentiated neuroendocrine tumors and carcinomas (67 vs. 27% of analyzed tissues by IHC) (13). In another study, Catena L. et al. showed that mTOR was expressed in 80% of patients who had poorly differentiated neuroendocrine carcinoma. They also found no relationship with tumor origin (pancreas, colon, lung, small bowel and others) or the rate of proliferation as determined by MIB-1 (>20% in all samples) (31). Molecular studies in SI-NEN cell lines (KRJ-I, H-STS) showed increased activation of AKT respective to normal Enterochromaffin (EC) cells that exhibited inferior expression of transcripts for AKT and mTORC1 as well as a lower level of Akt activation suggesting a neoplasia-related involvement of this pathway (32).

Jiao et al. analyzed the exomic sequences of 10 sporadic panNENs and screened the most frequently mutated genes in 58 pancreatic NENs. The mutations on MEN1 (44%), DAXX/ATRX (43%), TP53 (3%) were found. Notably, 15% of the tumors showed mutations in mTOR pathway-related genes (the onco-suppressor PTEN, the negative regulators TSC2, and PIK3CA, and the catalytic subunit of phosphatidylinositol 3-kinase) (33). In a clinical study, the patients with MEN-1, DAXX, and ATRX mutations had a median overall survival of 10 years in contrast with 60% of patients without mutation that died within 5 years of diagnosis. Based on the results of the study, the authors advanced the stratification of patients for treatment with mTOR inhibitors (34). In 2017 Scarpa et al. published a study on the whole genome sequencing of 98 pancreatic NETs in which they confirm the mTOR pathway activation in 15% of the analyzed samples. They identified mTOR pathway inhibitors alterations such as PTEN mutations (7.1%), TSC1 or TSC2 (2%). The study also proposed DEPDC5 inactivating mutations (2%) and EWSR1 fusion event as a novel mechanism of mTOR activation (14).

## Inhibitors of Akt-mTOR Signaling Pathway as Neuroendocrine Tumor Therapy

**Figure 2:**
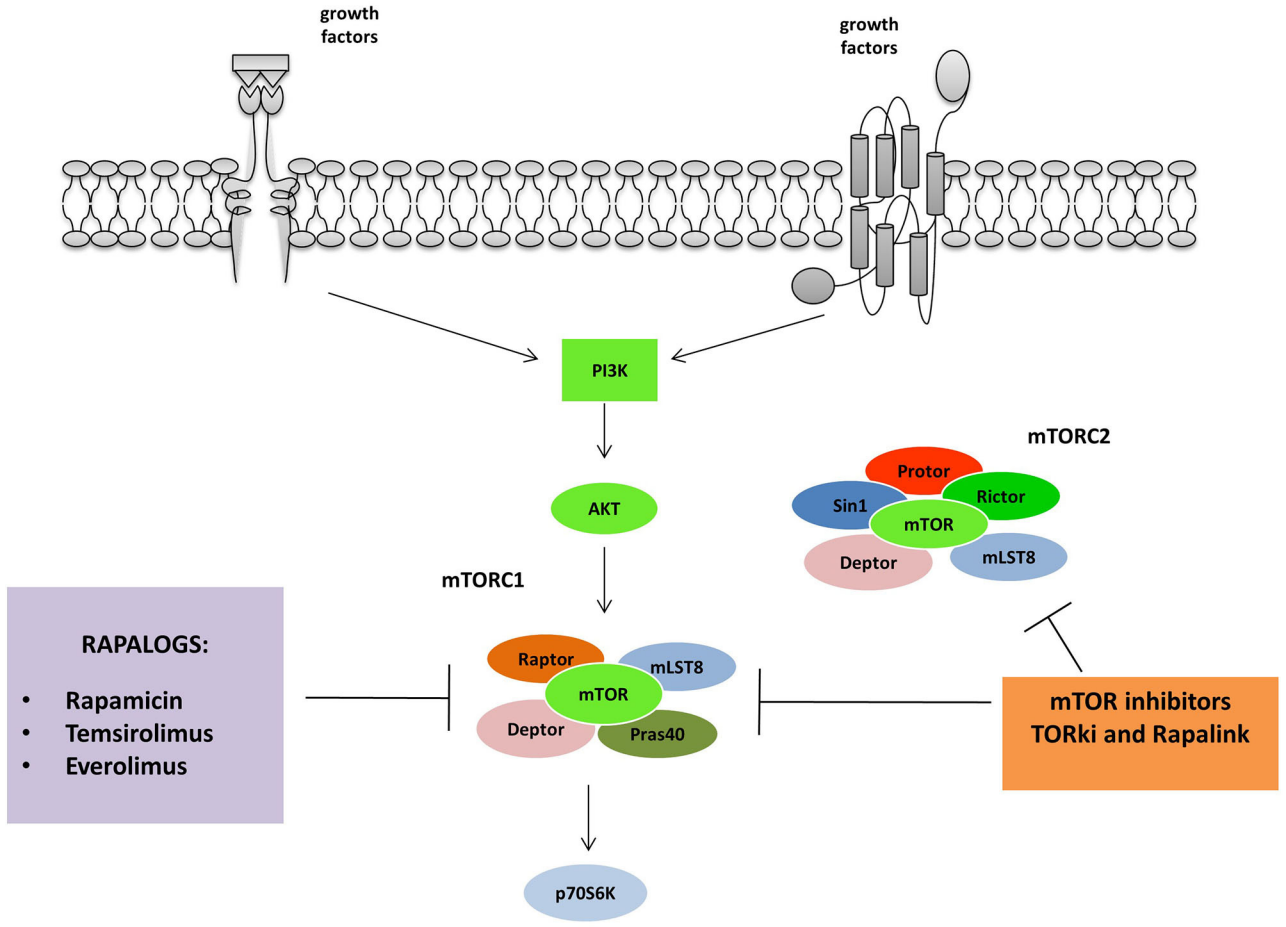
mTORC 1 and 2 are inhibited by different classes of mTOR inhibitors: Rapalogs are the first generation mTORC inhibitors able to induce a partial inhibition of mTORC1. The second and third generation act on both mTORC1 and 2. TORki blocks the ATP binding sites of the complexes, while Rapalinks act through blocking the ATP binding site and by the inhibition of the mTORC1.

mTOR has been the first node of the pathway to be targeted with a drug in tumors exhibiting phosphoinositide 3-kinase (PI3K) pathway mutation or activation (35, 36). First-generation of mTOR inhibitors includes Rapamycin (Sirolimus), an immunosuppressant agent identified as a fungicide isolated from the soil bacterium Streptomyces hygroscopicus (37). Derivatives of Rapamycin, referred to as rapalogs, (Temsirolimus, Everolimus, and ridaforolimus), function similarly to inhibit mTOR, although they have better efficacy and activity which optimizes clinical use. The Food and Drug Administration (FDA) firstly approved (Figure 2).

### Temsirolimus

Temsirolimus inhibits mTOR activity by binding the intracellular protein peptidyl-prolyl cis-trans isomerase FKBP1A (FKBP-12) (38). The inhibition results in a G1 growth arrest and in a blockade of the mTOR ability to phosphorylate S6K1 and the ribosomal protein S6, and in reduced levels of HIF-1α, HIF-2α, and VEGF expression. In a phase II trial, Duran et al. (39) evaluated the efficacy, safety, and pharmacodynamics of Temsirolimus amongst 37 patients with advanced neuroendocrine carcinoma (21 carcinoids and 15 islet cell carcinomas). Patients were treated with weekly intravenous doses of 25 mg of Temsirolimus and then evaluated on several outcomes, including tumor response rate, time to progression, adverse events, and overall survival. Data were analyzed with intention-to-treat modeling and revealed a response rate of 5.6% [95% confidence interval (CI), 0.6–18.7], respectively 4.8 and 6.7% in carcinoids and islet cell carcinomas. The median time to progression was 6 months. The 1-year survival rate was 71.5%. The study confirmed the inhibition of the phosphorylation of the ribosomal protein S6 in paired baseline and post-treatment biopsies (*p* = 0.02). With higher baseline levels of pS6, there was a non-significant trend toward a better response (*P* = 0.097). Higher baseline levels of phosphorylated mTOR were significantly correlated with a better response (*p* = 0.01).

On the contrary, after 2 weeks of treatment, an increase in the expression of pAKT and a decreased expression of phosphorylated mTOR were observed, both associated with increasing time to progression (*p* = 0.04 and *p* = 0.05, respectively). Given the low response rate, the authors concluded that Temsirolimus appears to have limited clinical utility as a single agent for patients with GEP-NENs. The study proposed evaluating Temsirolimus in combination with other targeted agents, for example, a multi-kinase inhibitor or an anti-angiogenic compound (39). A phase II trial involving 58 patients (56 eligible) was performed to investigate the efficacy of temsirolimus and bevacizumab association. Results showed an increased response rate (RR) of 41% exceeding the single-agent RRs measured by RECIST criteria and a PFS at 6 months of 79%. The therapy administrated to moderate-well-differentiated metastatic P-NETs showed substantial activity and no high toxicity since the most common adverse events were hypertension, fatigue, hyperglycemia and lymphopenia (40).

## Everolimus

### Single Therapy

Everolimus is a first-generation oral mTOR inhibitor approved by the US FDA and EMEA for the treatment of P-NENs. Everolimus similarly acts as Temsirolimus inhibiting mTOR kinase binding to FKBP-12 and reducing the activity of mTOR downstream effectors S6K1 and 4E-BP1 (41, 42). A phase I study involving 55 patients with advanced solid tumors, including NETs, evaluated Everolimus safety and pharmacodynamics. The trial aimed to establish an evidence-based dose and effective schedule for cancer treatment. A key criterion was the achievement of complete inhibition of mTOR dependent-signaling pathways on tumor and skin biopsies. Patients unresponsive to standard therapy were enrolled and treated with Everolimus with either 20, 50, or 70 mg weekly or 5 and 10 mg daily. Data suggested that Everolimus brought about both a dose- and schedule-dependent inhibition of the mTOR pathway. There was almost complete inhibition seen of the phosphorylated ribosomal protein S6 (*p* < 0.001) and further eIF4G (*p* < 0.001) expression at 10 mg/day and ≥50 mg/week. Although non-significant, there was a trend toward the reduction of phosphorylated 4E-BP1 expression (*p* = 0.058). Also, an overall increase in Akt phosphorylation, occurring in about 50% of patients, was observed (*p* = 0.006). This finding raises the question as to whether the upper regulation of pAKT may reduce the clinical effectiveness of the drug. The authors proposed a dose of 10 mg/day or 50 mg/week to be evaluated in further researches (43).

RADIANT-3 (44) was a phase III study aimed at evaluating Everolimus at 10 mg/day as monotherapy (*n* = 207) or placebo (*n* = 203), with a total sample size of 410 patients with progressive P-NENs, both in conjunction with best supportive care including the use of somatostatin analogs. This trial demonstrated 2.4 odds of improvement in median PFS (11.0 vs. 4.6 months; HR = 0.35; 95% CI: 0.27–0.45; *P* < 0.001) in the arm treated with Everolimus. The trial concluded that although the exact sequencing of therapies to treat of panNENs remains unclear, Everolimus can be advanced as effective in patients with prior chemotherapy or therapy-naïve prolonging PFS (44). RADIANT-4 involved 302 patients with advanced GI and Lung NET's. The Everolimus showed an increase in PFS of 7.1 months in respect to the placebo comparable along with disease stabilization similarly to the results obtained in the RADIANT 3 (45). In a prospective, randomized, pharmacokinetic, crossover trial comparing everolimus 10 mg once daily with 5 mg twice daily Verheijen et al. showed that switching everolimus from once daily to twice daily could reduce the toxicity and maintain treatment efficacy (46).

## Combination Therapy

### Somatostatin Analog (SSAs) Octreotide and Pasireotide

Octreotide is a first-generation SSA that is used to control the symptoms in NET's and exhibited tumor growth inhibitory function in metastatic well-differentiated midgut NET's (47). The first trial included 60 patients diagnosed with advanced low- to intermediate-grade GEP-NETs. Of these, 30 patients had carcinoid tumors, and 30 had islet cell carcinomas. All were treated with intramuscular octreotide LAR 30 mg every 28 days and oral Everolimus, 5 mg/day (patients 1 to 30) or 10 mg/day (patients 31 to 60) every 28 days. Overall response (OR) rate was 20%. In details, 70% of the patients showed stabilization of the disease, and 22% confirmed partial responses. The overall median progression-free survival (PFS) of patients treated with octreotide LAR and RAD001 was 60 weeks (95% CI, 54–66 weeks). Therefore, the trial showed that Everolimus, in combination with octreotide LAR, presented promising antitumour activity in patients with advanced NETs (48). The second phase II trial (RADIANT-1) assessed the antitumour activity of oral Everolimus at 10 mg daily in 115 patients with advanced pancreatic NETs who had disease progression during or after cytotoxic chemotherapy. The study confirmed the antitumour activity of Everolimus in panNENs in both groups, those receiving Everolimus alone (PFS was 9.7 months and ORR = 9.6%,) and Everolimus with Octreotide (PFS was 16.7 months and ORR = 4.4%) (49). Following the results of the two randomized phase II clinical trials, RADIANT-2 was planned. RADIANT-2 was a landmark and the most extensive study to have been conducted. RADIANT-2 involved 429 patients with progressive functional carcinoid tumors. The study was conducted to compare Everolimus, at a dose of 10 mg per day, plus octreotide LAR, 30 mg every 28 days, vs. placebo plus octreotide LAR at the same doses. In this trial, the primary endpoint was to evaluate PFS. PFS was 16.4 months on the Everolimus plus octreotide LAR arm vs. 11.3 months on the placebo plus octreotide LAR arm (hazard ratio = 0.77; 95% CI, 0.59–1.00; *p* = 0.026) (50). The results support the efficacy of Everolimus as an effective intervention for a broad spectrum of advanced neuroendocrine tumors. In a final analysis of the overall survival (OS) data from the RADIANT-II study, Pavel et al. showed that the median OS (95%CI) after 271 events was 29.2 months (23.8–35.9) for the everolimus arm and 35.2 months (30.0–44.7) for the placebo arm (HR, 1.17; 95% CI, 0.92–1.49) with no significant differences in OS between the two group (51). The ITMO group study was set up on 50 patients with different NETs. The results showed an objective response rate (ORR) of 18%; complete response in 4% of the patients and a partial response in 16% while 74% showed disease stabilization. Similarly to the RADIANT-2, the study suggests antitumour benefit in the use of Everolimus plus octreotide as a treatment in NETs, even if, the small number of patients included in the study must be considered in the data interpretation (52).

**Figure 3:**
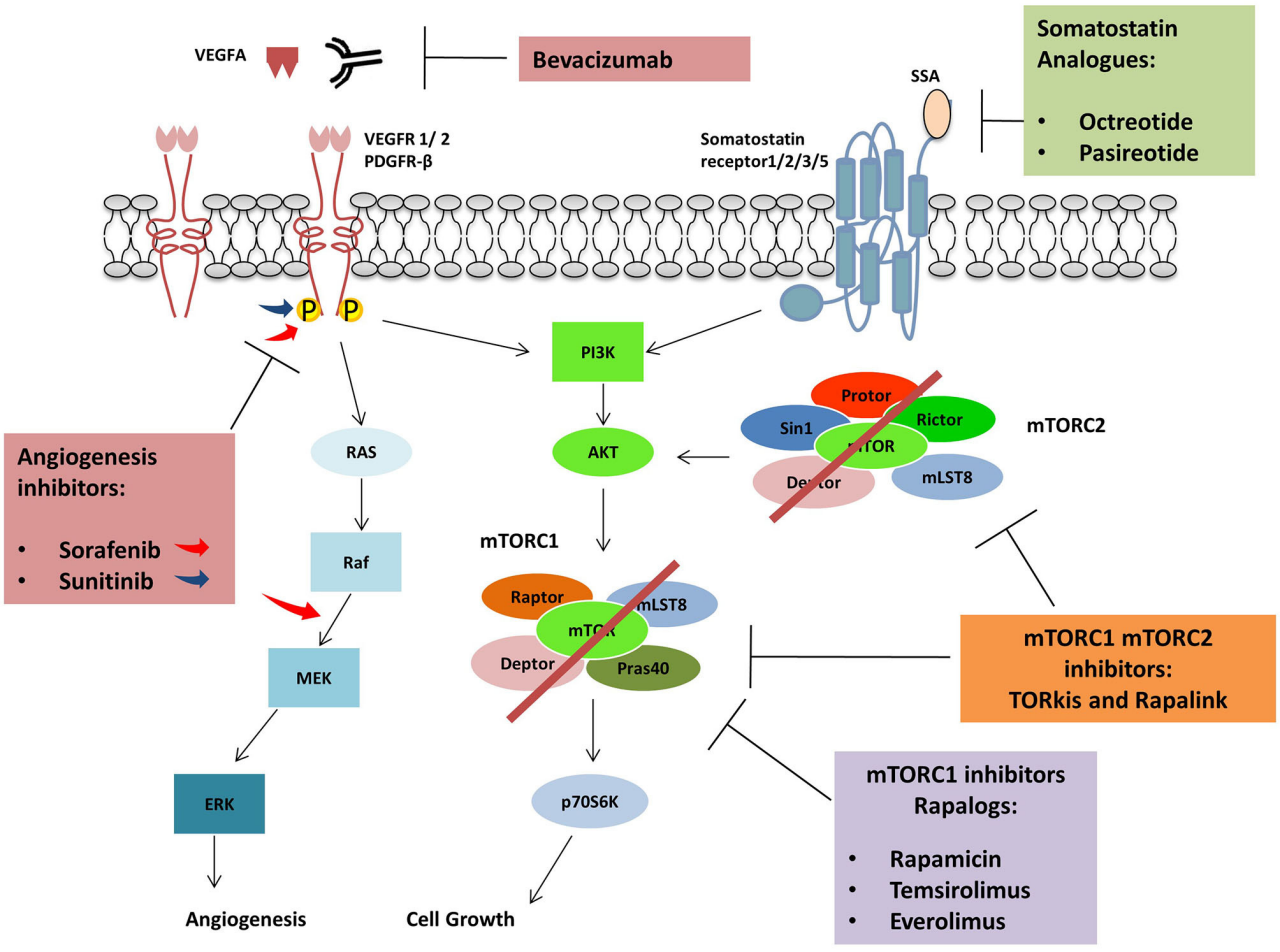
Agents used in combination with mTORC1 inhibitors: Protein kinase inhibitors, Sorafenib and Sunitinib are active against several tyrosine kinases receptors (RTKs) including VEGFR and PDGFR. Sorafenib can also inhibit RAF kinases. The monoclonal antibody Bevacizumab inhibits angiogenic pathway binding VEGFA and avoiding VEGFRs activation. Somatostatin analogs bind somatostatin receptors impairing cell growth while Rapalogues can inhibit mTORC1.

Pasireotide is a second-generation SSA, targeting the somatostatin receptor subtype 1,2,3 and 5 (53). In a randomized phase 2 study, Everolimus was administrated with Paoirreotide or in monotherapy to 160 NETs patients. However, no improvement of PFS was observed between the two groups, and no benefit was found in the use of drugs combination (54). Contrarily, another study made on 21 NETs patients treated with increasing doses of Pasireotide (until 60 mg monthly) and Everolimus (5–10 mg daily) confirmed the antitumour activity (81% of patient experienced a grade of tumor regression) and the tolerability in term of side effects of this therapy (55). The combination of selective internal radioembolisation (SIRT), Everolimus, and Pasireotide showed encouraging results in a study involving 13 NETs patients (median progression-free survival 18.6 months and overall survival 46.3 months) at a low level of toxicity (53) (Figure 3).

## Everolimus and Anti-Angiogenetic

NETs are high vascularised tumors, and this observation laid the groundwork/basis for the investigation of a synergistic effect through combined targeting of mTOR pathway and VEGF (56). A potent anti-angiogenic and antivascular effect were observed after the treatment with Everolimus of various solid tumors. The mechanism was different from those found with VEGFR targeting agents. Everolimus inhibited the proliferation of human endothelial cells and impaired VEGF release from cancer cells while VEGFR inhibitor PTK/ZK inhibited endothelial cell migration and vascular permeability. The results suggested the use of rapalog in combination with VEGF inhibitors as an effective therapeutic strategy to obtain a stronger diminishing of tumor vascularisation (57). Sorafenib is a drug inhibiting PDGFRB, and VEGFR2 also found to have modest activity in phase II study on NET's patients (58). In a phase I trial, 21 patients were treated with 10 mg daily Everolimus and two different doses of sorafenib (400 and 600 mg daily), the maximum tolerated dose (MTD) was established in 400 mg per day. A partial response was observed in one patient while a limited tumor regression in 13 out of 21 patients (62%) (56). Furthermore, the combination of Everolimus and Sunitinib to target both the PI3K/Akt/mTOR and VEGF signaling was evaluated as a therapy for different cancers. However, the treatment showed significant acute toxicity (59). Sunitinib is a multitarget tyrosine kinase inhibitor directed against different receptors such as VEGF-R1/2/3, PDGF-R α/β, Stem cell factor receptor (c-KIT-R). Also, colony-stimulating factor 1 receptor (CSF1-R), FML like tyrosine kinase three receptor (FLT3-R) and glial cell line-derived neurotrophic factor receptor (RET) (60). The drug showed comparable efficacy for Everolimus as first-line therapy in phase II study (61). Sequential administration was studied in 31 patients as high toxicity when the two drugs were simultaneously administrated. The results showed good tolerability with no differences in median PFS between the two groups (Everolimus followed by sunitinib, 36.5 months vs. Sunitinib followed by Everolimus, 31.6 months) (62). Another drug investigated to find a synergistic effect with Everolimus and to impair vascularisation in NETs was Bevacizumab in a randomized phase 2 study on 150 patients. The combination of the two drugs showed an increase of PFS (16.7 vs. 14 months), but also the adverse events were more frequent in patients receiving both drugs (63).

### Everolimus Plus Target and Radionuclide-Therapy

Everolimus was also tested in phase I/II study, in combination with temozolomide in 43 pancreatic NETs, 40% of the patients (40 evaluable patients) had a partial response with median progression-free survival (PFS) of 15.4 months (64). In a phase I study (NETTLE) involving 16 NETs patients the toxicity of Everolimus in combination with PRRT (Lutetium-177-octreotate) was investigated and Everolimus 7.5 mg per day appeared to be well-tolerated (65).

### Second and Third-Generation mTOR Inhibitors

To overcome the resistance phenomenon and to have a complete inhibition of the mTOR pathway, second-generation inhibitors were synthesized. These compounds are called TORkis and act binding the ATP binding site “of mTOR kinase pocket.” Differently from the rapalogs, these molecules ensure a complete block of both MTORC1 and 2 preventing the Akt phosphorylation due to MTORC2 and avoiding the resistance observed in rapalogs. Different TORkis were synthesized and showed promising results in pre-clinical studies. PP242 and the derived compound MLN0128, the quinolone-derived torin1 and 2, QSI-027, ku0063792 and Ku-0068650 showed a high antiproliferative power. From the latter derived AZD8055 and AZD2014, which was primarily tested in clinical trials even in combination with other therapeutic agents in different solid tumors (66).

The mTORC1/2 kinase inhibitor named CC-223 was tested in a phase 1/2 study involving metastatic non-pancreatic GI-NETs patients treated with SSA who had failed treatment. The drug showed efficacy in induce tumor regression and carcinoid syndrome symptoms controls and led to an Improvement of median PFS (19.5 months) superior to Everolimus alone (PFS 11.0 months) (44, 67). The CC-223 safety profile was found to be comparable to currently approved mTOR inhibitors, and toxicity was well-managed by dose adjustments or treatments (67).

Third generation mTOR inhibitors were studied to address the treatment resistance issues found in the use of the rapalogs and TORkis (68). The new compounds are called RAPAlink since they are made by the conjugation of TORkis, having high affinity for ATP binding site of both MTORC1/2 and Rapamycin having the FKB12-dependent mechanism to block MTORC1. These compounds sowed increased and durable inhibitory action compared to the first and second-generation inhibitors and ability in crossing BBB in glioblastoma *in vitro* and *in vivo* (69). An *in vitro* study on the resistance to first and second-generation mTOR inhibitors showed the development of mutations in FKBP-12 (FRB domain) in Rapalogs resistant cells and mutations increasing intrinsic kinase activity of mTOR in TORkis resistance. These mechanisms have been overcome by the use of Rapalink able to establish a bivalent interaction of the two-binding site (68). Sapanisertib is an inhibitor of raptor-mTOR, and rictor-mTOR tested in several solid tumors (70). In a patient-derived xenograft model of PNET (PDX-PNET) the majority if everolimus-resistant PDX-PNETs responded to sapanisertib (71).

## GEP-NENs Treatment Resistance and Future Approaches (Rapalogs Resistance, Molecular Mechanisms)

**Figure 4:**
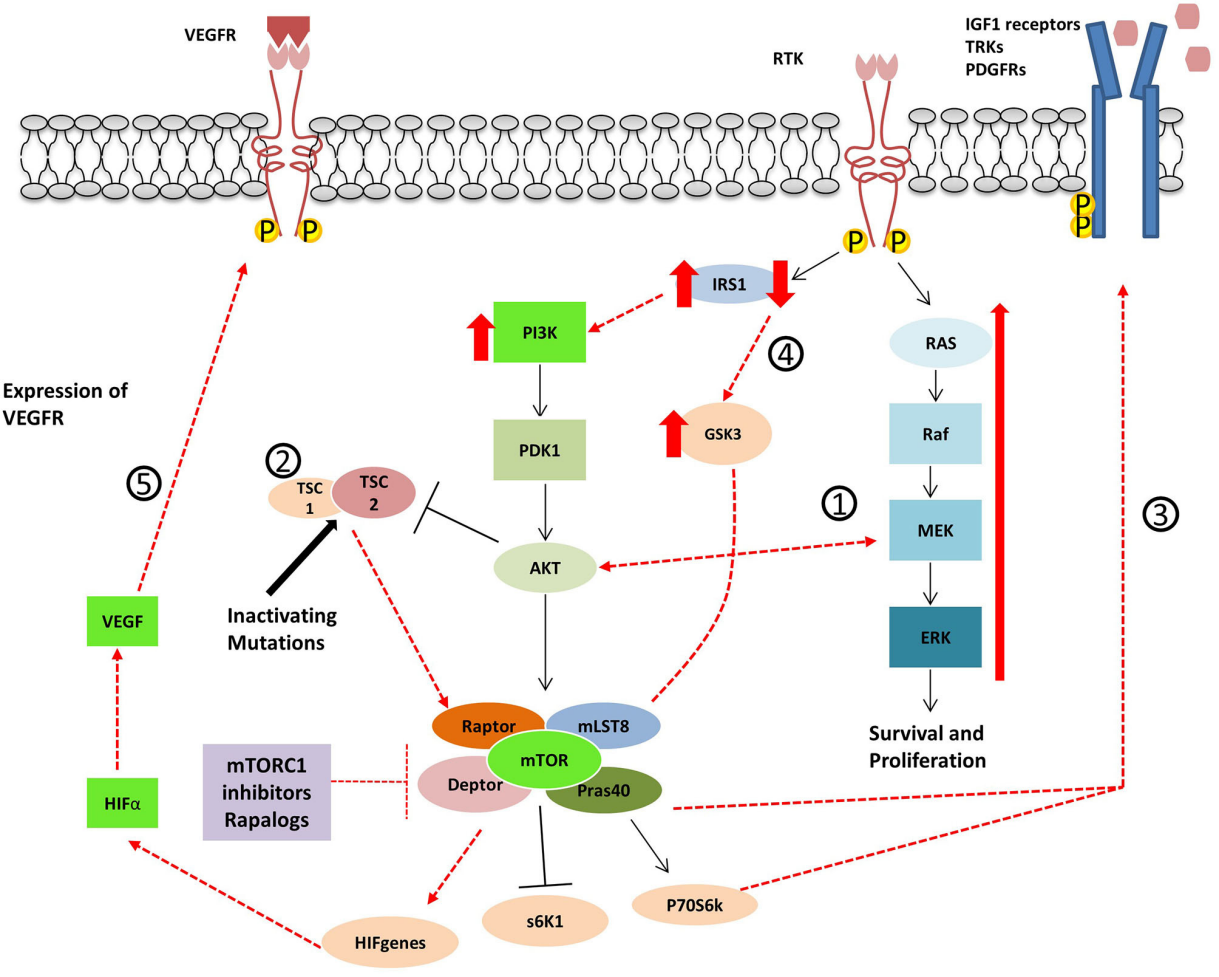
Proposed mechanisms for resistance to Rapalogues in NET. 1- Inhibition of mTOR results in PI3k-Akt-mTOR pathway reactivation and MAPK pathway activation. 2- Inactivating mutations in TSC1/2 cause the inactivation of the TSC1/TSC2 protein complex leading to mTOR hyperactivation. 3- mTOR inhibitor treatment cause an increase in tyrosine kinase receptors and growth factor secretion. 4- GSK3 over-expression accompanied by the decrease of IRS-1 protein leads to decreased autophagy and cell resistance to Everolimus. 5- The up-regulation of angiogenic factors mTOR-independent or the re-expression of HIFα.

GEP-NENs develop resistance to treatment, not only to standard target therapy and SSA but also to novel agents. After long-term exposition to prolonged targeted inhibition of a single pathway, cancer cells acquire therapeutic resistance activating alternative or compensatory pathways. The PI3K-Akt-mTOR and Ras/MAPK pathway are connected at multiple levels, and both can be mutuality activated or inhibited (72, 73). In other words, activation of the mTORC1 leads to PI3K and MAPK inhibition via a negative feedback loop system, and inhibition of mTOR, inversely, results in reactivation of PI3k-Akt-mTOR pathway and MAPK pathway. The resistance of antitumour effects of mTOR inhibitors is explored in most the studies investigating the PI3K/Akt pathway. However, several studies showed that mTOR inhibition resulted in an activation of the MEK/ERK cascade through a PI3K-dependent feedback loop (32, 74–76). The phenomenon may contribute to explain the escape of drug efficacy. Thus, combination therapies of mTOR inhibitors with MEK inhibitors have been proposed as an alternative mechanism to inhibit both pathways and overcome tumor resistance (77) (Figure 4).

Carracedo et al. showed that tumor samples were taken from patients with biopsy-accessible solid tumors of advanced disease and treated with RAD001. The study demonstrated robust activation of the MAPK pathway at specific doses and related to the administration schedule. The researchers also described a rapamycin-induced ERK/MAPK activation in both normal cells and cancer cells lines based on an S6K/PI3K/Ras pathway (74). Mi et al. evaluated the combinatorial inhibition of mTOR and MAPK pathway in mouse Tsc2 knockout cells by administrating both Rapamycin and MEK1/2 inhibitor, PD98059. The mutations in TSC1 or TSC2 result in the inactivation of the TSC1/TSC2 protein complex that leads to hyperactivation of mTOR, causing uncontrolled cell growth and proliferation. The inhibitory effects on proliferation in Tsc2 deficient cells were higher using the combinatorial approach (75). Ziztmann et al. studied the complex interplay between PI3K/Akt/mTOR pathway and MAPK pathway using different drugs combination in different human NET cell lines. The study showed that cells develop a mechanism of escape when using a single agent target pathway also through compensatory induction of AKT. They noted that the dual inhibition of mTOR (Everolimus) and PI3K (NVP-BEZ235) had a more significant effect than the single inhibition of mTOR in cell lines (78). However, two trials on NVP-BEZ235 were early stopped due to unmet statistical endpoint or intolerable toxicity (79).

The resistance to mTOR inhibitors has also been proposed through other potential mechanisms. O'Reilly et al. (80) reported that mTOR inhibition induces insulin receptor substrate-1 (IRS-1) expression resulting in AKT activation both in cancer cell lines and in tumor tissues treated with RAD001. AKT activation after mTORC1 inhibition has also been demonstrated depending on upregulation of RTKs such as PDGFRs (81, 82). It has been shown that SI-NEN cell lines escape from mTOR inhibitor treatment through dual feedback activation of Akt and ERK1/2 *via* an increase in tyrosine kinase receptors and growth factor secretion. Concurrent therapy with octreotide failed to overcome the escape phenomenon suggesting dual targeting of PI3K/Akt/mTOR pathway and MAPK pathway as an alternative method to reverse feedback cross-activation (32).

From an *in vitro* study on everolimus resistant panNET cell lines (BON1 RR1, BON1 RR2) Gsk3 was found to be dysregulated. In these models, the GSK3 hyperactivation was associated with reduced IRS-1 protein levels, decreased autophagy and cell cycle arrest in G1 phase due to CDK1 (cdc2) reduced expression. Interestingly, A PI3Kα-inhibitor (BLY719) used in combination with everolimus was able to re-establish the everolimus sensitivity (83).

Pro-angiogenic factors upregulation can also be involved in rapalogs resistance since mTOR inhibition has been proven to have a direct and indirect anti-angiogenetic effect (57).

NETs, especially those well-differentiated, are high vascularised tumors due to the significant HIFα up-regulation which may arise by genetic alteration of the VHL protein and to the tumor microenvironment (84, 85) PI3K/AKT/mTOR pathway regulates the angiogenesis in NETs modulating (84–87) The Aurora Kinase A (AKURA) overexpression has been observed in everolimus resistant GI adenocarcinoma cell lines. This protein can mediate elF4E phosphorylation and increase c-Myc levels. The AURKA-EIF4E-c-MYC axis can be an alternative target for everolimus resistant tumors (86, 88, 89).

Several studies have been performed to found predictive biomarkers allowing the stratification of patients that may benefit from therapy with mTOR inhibitors. Recently, mRNA-based evaluation (NETest) performed on the tumor has proven to be a useful biomarker for NETs. NETest is a gene panel analyzed from a liquid biopsy and represents an innovative non-invasive approach to disease progression evaluation that better performs in respect to conventional biomarkers such as CgA (90).

CgA and neuron-specific enolase (NSE) have been proposed as markers of (91) From the RADIANT1 trial CgA > x2 ULN, two times higher than normal levels (36,4 ng/mL) is linked to the worst prognosis and shorter PFS in panNET's. Similarly, NSE >2xULN were associated with a shorter PFS. Although baseline CgA and NSE levels failed to predict mTOR therapy responsiveness, prospective analysis on a large number of patients showed a correlation between an early decreasing in CgA or NSE levels in response to Everolimus treatment (>30% decrease from baseline or normalization after 4 weeks) and a significant improvement of PFS (48). Furthermore, 5-hydroxyinoleacetic acid (5-HIAA) was found to be related to an increase in PFS in patients receiving Everolimus (92, 93).

Correlations between rapalogs sensitivity and the levels or activation status of the mTOR pathway signaling components were found (94). Baseline Phosphorylation of mTOR signaling molecules has been related to the worst outcome in NET's patients but also with a better response to Everolimus (95). For this reason, the increase of phosphorylated Akt was proposed as a biomarker in case of reduced PTEN expression to individuate tumors responding to mTOR inhibitors. Akt phosphorylation (S473 and T308) was more likely found in patients responding to rapamycin than non-responders (96). However, as discussed in the previous chapter, Akt was also found to be phosphorylated (ser473) in case of rapalogs resistance due to MTORC2 activation (97).

PTEN mutations were investigated as a possible predictive biomarker, and several studies pointed out that PTEN null cells, as well as xenograft models with reduced PTEN activity, were more sensitive to rapalogs (prostate cancer) (94). PTEN mutations which are related to the increase of the mTOR pathway activation has been found in different diseases as well as in NET's (14).

High sensitivity to rapalogs was observed in *in vitro* and *in vivo* NET's models with mutated PIK3CA/PTEN and high p-Akt levels (96). Interestingly, response to everolimus was lost when PIK3CA mutation occurred together with KRAS mutation. However, everolimus sensitivity was re-established in HCT116 cells in which the KRAS D13 mutant allele had been genetically deleted by homologous recombination (98). Single nucleotide polymorphism is investigated in cancer and in particular, the SNP GFR4-G388R was observed in panNET patients. The fibroblast growth factor receptor 4 (FGFR4) plays a role in mitogenesis and angiogenesis and the presence of an arginine instead glycine in the codon 388 was related with shorter PFS especially in heterozygous patients compared to homozygous for the SNP (PFS 4.8 vs. 16.6 months, respectively; OS of 9.3 vs. 40 months, respectively). Also, the SNP was found to be related to a higher risk of liver metastasis and was present in patients not responding to everolimus (99). Contrarily, Cros et al. who studied the FGFR4 polymorphism (G388R) on 41 patients with NET's did not found a correlation between PFS and the presence of SNP (100). The inactivating PHLPP2-L1016S SNP was investigated as a possible predictive marker and was found to be associated with a reduced PFS in extra-pancreatic NET's patients treated with Everolimus. PFS was 16.8 months in wild type PHLPP2 patients vs. 7.7 months in those harboring SNP. Overall survival and response rate were not affected by the SNP presence. The results suggested that wild type PHLPP2 patients may benefit more from everolimus therapy. Interestingly, PLPP2 is known as a regulator of AKT that in turn, activate the mTOR pathway (101).

Falletta et al. successfully used patients derived primary cultures as a tool to predict the sensitivity to everolimus treatment. The study showed that IGF1 is related to everolimus antiproliferative effect only in patients with higher phosphorylated IGF1R levels, p-Akt, p-mTOR, p-4EBP1 and higher Ki67 index (responders) compared to non-responders to mTOR inhibitors (102).

Another possible predictive biomarker for rapalogs sensitivity is the presence of mTOR activating mutations. A study showed the presence of missense mutations in 400 oncologic patients' samples with different cancer subtypes. The mutations were present in 6 various sites but most frequently in the C-terminal region of the protein. In a subgroup of these samples, the hyperactivation of mTOR was due to the impairment of mTOR-DEPTOR inhibitor binding. The activating mutations observed in cell culture and xenografts were linked to an increased sensitivity to Rapamicin (103). Contrarily, activating mutations of mTOR has been observed in cell resistant to TORkis targeting the ATP binding pocket (68).

### Meta-Analysis

In a meta-analysis including studies performed on 1908 NET's patients, target therapies were found to be effective and improve PFS (hazard ratio = 0.59, 95% CI:0.42–0.84; *P* = 0.003) in particular in pancreatic NET's patients (HR = 0.49 95% CI: 0.29–0.83) than in non-pancreatic NET's (HR = 0.71 95% CI: 0.49–1.02). Target therapies with Everolimus and with sunitinib (monotherapies) or Everolimus and octreotide were found effective in pan NETs (104).

Recently, a meta-analysis comprising 3,895 cases of NETs evaluated the most promising therapies in panNET's. Everolimus as single therapy (0,82 P score)/(hazard ratio [HR], 0.35 [95% CI, 0.28–0.45]) appeared to be the most effective treatment followed by SSA combined with Everolimus (0,73 P score)/ (HR, 0.35 [95% CI, 0.25–0.51]). The combination therapy with interferon and SSA (P score 0.71) was also found to be effective followed by the monotherapies interferon (P score 0.62), SSA (0.54 P score)/(HR, 0.46 [95% CI, 0.33–0.66]), sunitinib (0.39 P score), Dactolisib (0.6 P score), and placebo (0.13 P score).

In 8 studies assessing the PFS after nine different therapies, Everolimus showed high effectiveness in panNETs, especially in combination with SSA (0.72 P score) as well as in monotherapy (0,72 P score). The combination of the mTOR inhibitor with bevacizumab and SSA showed lower efficacy (0.44 P score)/ (HR, 0.44 [95% CI, 0.26–0.75]). The best performing therapy improving PFS was the combination of SSA with interferon (0.77 P score)/(HR, 0.31 [95% CI, 0.13-0.71]. Differently, in GI-NET's the most efficient therapy in disease control was the combination of bevacizumab with SSA (0.93 P score)/ (HR, 0.22 [95% CI, 0.05–0.99]) followed by ^177^Lu-dototate and SSA (0.92 P score)/(HR, 0.08 [95% CI, 0.03–0.26] and interferon plus SSA (0.66 P score)/(HR, 0.27 [95% CI, 0.07–0.96]). Everolimus with SSA (0.52 P score)/ (HR, 0.31 [95% CI, 0.11–0.90]) was found to be less effective in GI-NETs than in panNETs. Furthermore, Everolimus as monotherapy resulted to be effective comparably to SSA alone (0.39/ (HR 0.48 [95% Cl, 0.20–1.13]) vs. 0.4/(HR, 0.40 [95% CI, 0.21–0.78]) P scores, respectively) (105).

Studies on the quality of life and adverse events (AE) showed that the combination SSA with Everolimus reported one of the highest numbers of AE (82.7% of the patient), with 68% of these being grade 3–4. Similarly, Everolimus used in monotherapy caused AE in 92.1% of the patients, but 59.3% of grade 3–4. Among the therapies showing good efficacy, SSA in combination with ^177^Lu-dototate showed a better profile in term of toxicity in respect to Everolimus (94% AE, 41% grade 3–4). Interferon and SSA caused AE in 21% of the patient and a small percentage of a grade 3–4 (3%) while SSA alone caused AE in 69.8% of the cases and 20.9% of AE of grades 3–4 (105). A meta-analysis of individual patient data showed that a 2-fold increase in Everolimus Cmin delayed NET disease progression with improved tumor size reduction. However, the protocol increased the risk of high-grade toxicity, mainly with a high number of pulmonary, metabolic and stomatitis events (106). Mujica-Mota et al. evaluated the clinical effectiveness of three interventions (everolimus, lutetium-^177^ DOTATATE, and Sunitibib). The primary limitation was that there was no RCT comparing lutetium-^177^ DOTATATE with the other treatments. The authors concluded that based on NICE guidelines, only sunitinib could be considered cost-effectiveness in England and Wales (107).

## Conclusions

Treatment of advanced neuroendocrine neoplasms is an ongoing clinical challenge. RCTs are mainly focused on treatments with Everolimus and SSAs (Table 1). Everolimus administration in advanced NET's demonstrates its efficacy and high tolerability both as monotherapy and in combination with other drugs. However, the use of Everolimus has been known to lead to resistance due to several mechanisms such as feedback activation of alternative pathways, inactivation of protein kinases, and deregulation of the downstream mTOR components (108, 109) Next-generation mTOR inhibitors have been studied to avoid the mechanisms of resistance and reduce the drug toxicity (85). Levels of CgA and NSE can predict outcomes in patients with advanced pNETs treated with everolimus, and other circulating biomarkers have been studied. There are several limitations with treatment outcomes (e.g., lack of benefit in OS from mTOR inhibitors) and biomarkers clinical application (e.g., small study sample size). However, the present review suggests that a range of combination therapies associated with the use of predictive biomarker is available for NET patients. Therefore, new emerging compounds such as second and third-generation mTOR inhibitors and anti-angiogenetic drugs should be tested in RCTs.

**Table 1:** Clinical trials on mTOR inhibitors in neuroendocrine tumor.

**Study**	**Patients**	**Type of tumor**	**Progressive metastic disease**	**Drug**	**Combination therapy**	**Median OS (months)**	**Response rate**	**Median Progression free survival (PFS, months)**	**Molecular markers analyzed**	**References**
Phase-II	37	Carcinoid 21 islet cell carcinoma 15	yes	Temsirolimus 25 mg/w	no	Not reached	5.6%	6 (TTP)	PTEN, p53, pAKT, pS6, pmTOR.	Duran et al. (45)
Phase-II	56	Well or moderately differentiated pancreatic neuroendocrine tumors	yes	Temsirolimus 25 mg/week	bevacizumab 25 mg/kg (once every 2 weeks)	34.0	41%	13.2	CgA Circulating hormones level	Hobday et al. (46)
Phase-I	55	Neuroendocrine neoplasms	Yes	Everolimus 20, 50, 70mg/w or 5,10 mg/d	No	-	-	-	pAKT and AKT, p4E-BP1 and 4EBP1, pS6, and S6	Tabernero et al. (48)
Phase-II	30 30	Low-to intermediate grade neuroendocrine neoplasms	Ns	Everolimus 5 mg/d everolimus 10 mg/d	octLAR 30 mg every 28 d	Not reached	20%	12.5 18	Ki-67	Yao et al. (52)
Phase-II	50	Advanced well-differentiated NETs	Yes	Everolimus 10 mg/d	OctLAR 30 mg every 28 d	Not reached	18%	-	CgA	Bajetta et al. (55)
RADIANT-1, Phase-II	115 45	Low-to intermediate grade pancreaticneuroendocrineneoplasms	Yes No	Everolimus 10 mg/d everolimus 10 mg/d	No octLAR 30 mg every 28 d	24.9 not reached	9.6% 4.4%	9.7 16.7	CgA NSE	Yao et al. (53)
RADIANT-2, phase-III	216 213	Low-to intermediate grade neuroendocrine neoplasms	Yes	Everolimus 10 mg/d placebo	octLAR 30 mg every 28 d octLAR 30 mg every 28 d	Not reached	-	16.4 11.3	CgA	Pavel et al. (54)
RADIANT-3, phase-III	207 203	Low-to intermediate grade pancreatic neuroendocrine tumors	yes	Everolimus 10 mg/d placebo	Best supportive care	Not reached	5% 2%	11.0 4.6	-	Yao et al. (49)
RADIANT-4 phase-III	205 97	Advanced, progressive, well-differentiated, non-functional lung or gastrointestinal neuroendocrine tumors	yes	Everolimus 10 mg/d Placebo	Best supportive care	23.7 16.5	64% 26%	11.0 3.9	-	Yao et al. (50)
Phase-I	21	Advanced neuroendocrine tumors	ns	Everolimus 5, 10 mg/d	Pasireotide s.c. 600, 900, 1,200 μg Pasireotide LAR 40,60,80 mg monthly	-	81%	-	Aminotransferase alanine-aminotransferase serum creatinine neutrophil count CgA.	Chan et al. (58)
Phase-II	160	Well-differentiated neuroendocrine tumors	yes	Everolimus 10 mg/d	PasireotideLAR 60 mg every 28 d	22.6	20%	16.8	CgA NSE IGF-1/2, IGFBP-2/3	Kulke et al. (57)
Phase I-II	7 phase I 36 phase II	Advanced pancreatic neuroendocrine tumors	ns	Everolimus 5 mg/d Everolimus 10 mg/d	Temozolomide 150 mg/m^2^ Temozolomide 150 mg/m^2^ (days 1 to 7 and days 15 to 21 of a 28-days cycle).	Not reached	40%	15.4	CgA	Chan et al. (66)
Phase I	13	Moderately or well-differentiated neuroendocrine tumors	Yes	Everolimus 2.5, 5, 10 mg/d	Pasireotide s.c. 600 μg twice daily Along with SIRT yttrium-90 on days 9 and 37	46.3	46%	18.6	Angiopoietin 1/2, bfgf, collagen V, IGF1/2, IGFBP, IL8, PGF, VEGFR2, CgA, prolactin, HGF.	Kim et al. (56)
Phase I	21	Locally unresectable metastatic carcinoid and pancreatic neuroendocrine tumors	Yes	Everolimus 10 mg/d	Sorafenib 400 mg/d Sorafenib 600 mg/d	-	62%	Pf-6 months 79%	CgA	Chan et al. (59)
NETTLE Phase I	16	Advanced unresectable progressive well-differentiated GEP-NETs	No	Everolimus 5, 7.5, 10 mg/d	PRRT ^177^Lu-octreotate 240 mg every 8 weeks	57	44%	-	CgA. urinary 5-HIAA	Claringbold et al. (67)
Phase-II	150	Advanced pancreatic neuroendocrine tumors		Everolimus 10 mg/d and octreotide acetate 20 mg once	Bevacizumab 10 mg/kg every 15 days	36.7	31%	16.7	-	Kulke et al. (65)

*w, weekly; d, daily; ns, not specified; oct, octreotide; CgA, chromogranin A; NSE, neuron-specific enolase; bfgf, basic fibroblast growth factor; IGF, insulin like growth factor; pgf, placental growth factor; VEGFR, endothelial growth factor receptor; HGF, hepatocyte growth factor; 5-HIAA, 5-hydroxyindoleacetic acid*.

## Author Contributions

SZ, FG and GB conceptualized the study. SZ, FG and SR identified relevant literature. SZ, FG, GB and SR wrote the manuscript. All authors contributed to the article and approved the submitted version.

## Conflict of Interest

The authors declare that the research was conducted in the absence of any commercial or financial relationships that could be construed as a potential conflict of interest.

## References

[1] Modlin IM Lye KD Kidd M. A 5-decade analysis of 13,715 carcinoid tumors. Cancer. (2003) 97:934–59. 10.1002/cncr.11105 12569593

[2] Rehfeld JF. The new biology of gastrointestinal hormones. Physiol Rev. (1998) 78:1087–108. 10.1152/physrev.1998.78.4.1087 9790570

[3] Anlauf M. Neuroendocrine neoplasms of the gastroenteropancreatic system : pathology and classification. Horm Metab Res. (2011) 43:825–31. 10.1055/s-0031-1291307 22105474

[4] Lawrence B Gustafsson BI Chan A Svejda B Kidd M The epidemiology of gastroenteropancreatic neuroendocrine tumors carcinoid epidemiology incidence neuroendocrine tumor. Endocrinol Metab Clin. (2011) 40:1–18. 10.1530/ERC-13-0125 21349409

[5] Modlin IM Champaneria MC Chan AKCA Kidd M. A three-decade analysis of 3,911 small intestinal neuroendocrine tumors: the rapid pace of no progress. Am J Gastroenterol. (2007) 102:1464–73. 10.1111/j.1572-0241.2007.01185.x 17391319

[6] Modlin IM Oberg K Chung DC Jensen RT Herder Wouter W de Thakker RV Gastroenteropancreatic neuroendocrine tumors. Lancet Oncol. (2008) 9:61–72. 10.1016/S1470-2045(07)70410-2 18177818

[7] Warner PRR. Enteroendocrine tumors other than carcinoid: a review of significant advances. Gastroenterology. (2005) 128:1668–84. 10.1053/j.gastro.2005.03.078 15887158

[8] Frilling A Akerstrom G Falconi M Pavel M Ramos J Kidd M Modlin I. Neuroendocrine tumor disease : an evolving landscape. Endocr Relat Cancer. (2012) 19:163–85. 10.1530/ERC-12-0024 22645227

[9] Pellikka PAAJTBKKJBS, JACHCP, and LKK. Carcinoid heart disease. Clinical and echocardiographic spectrum in 74 patients. Circulation. (2015) 87:1188–96. 10.1161/01.CIR.87.4.1188 7681733

[10] Cives M Strosberg JR. Gastroenteropancreatic neuroendocrine tumors. CA Cancer J Clin. (2018) 26:29–36. 10.3322/caac.21493 30295930

[11] Pavel M Öberg K Falconi M Krenning EP Sundin A Perren A. Gastroenteropancreatic neuroendocrine neoplasms: ESMO Clinical Practice Guidelines for diagnosis, treatment and follow-up. Ann Oncol. (2020) 31:844–60. 10.1016/j.annonc.2020.03.304 32272208

[12] Marinoni I Kurrer AS Vassella E Dettmer M Rudolph T Banz V. Loss of DAXX and ATRX are associated with chromosome. Gastroenterology. (2014) 146:453–60.e5. 10.1053/j.gastro.2013.10.020 24148618

[13] Shida T Kishimoto T Furuya M Nikaido T Koda K Takano S. Expression of an activated mammalian target of rapamycin (mTOR) in gastroenteropancreatic neuroendocrine tumors. Cancer Chemother Pharmacol. (2010) 65:889–93. 10.1007/s00280-009-1094-6 19657638

[14] Scarpa A Chang DK Nones K Corbo V Patch A-M Bailey P. Whole-genome landscape of pancreatic neuroendocrine tumours. Nature. (2017) 543:65–71. 10.1038/nature21063 28199314

[15] Leung R Lang B Wong H Chiu J Yat WK Shek T. Advances in the systemic treatment of neuroendocrine tumors in the era of molecular therapy. Anticancer Agents Med Chem. (2013) 13:382–8. 10.2174/1871520611313030002 23092266

[16] Willems L. PI3K and mTOR signaling pathways in cancer: new data on targeted therapies. Curr Oncol Rep. (2012) 14:129–38. 10.1007/s11912-012-0227-y 22350330

[17] Davis WJ Lehmann PZ Li W. Nuclear PI3K signaling in cell growth and tumorigenesis. Front Cell Dev Biol. (2015) 3:1–14. 10.3389/fcell.2015.00024 25918701 PMC4394695

[18] Zhang X Vadas O Perisic O Anderson KE Clark J Hawkins PT. Structure of lipid kinase p110b/p85b elucidates an unusual SH2-domain-mediated inhibitory mechanism. Mol Cell. (2011) 41:567–78. 10.1016/j.molcel.2011.01.026 21362552 PMC3670040

[19] Echeverria I Liu Y Gabelli SB Amzel LM. Oncogenic mutations weaken the interactions that stabilize the p110 a—p85 a heterodimer isn phosphatidylinositol 3—kinase a. FEBS J. (2015) 282:3528–42. 10.1111/febs.13365 26122737 PMC4573839

[20] Payne SN Maher ME Tran NH Hey V De Foley TM Yueh AE. PIK3CA mutations can initiate pancreatic tumorigenesis and are targetable with PI3K inhibitors. Oncogenesis. (2015) 4:e169–10. 10.1038/oncsis.2015.28 26436951 PMC4632089

[21] Hassan B Akcakanat A Holder AM Meric-Bernstam F. Targeting the PI3-kinase/Akt/mTOR signaling pathway. Surg Oncol Clin N Am. (2013) 22:641–64. 10.1016/j.soc.2013.06.008 24012393 PMC3811932

[22] Moschetta M Reale A Marasco C Vacca A. CMR. Therapeutic targeting of the mTOR-signaling pathway in cancer: benefits and limitations. Br J Pharmacol. (2014) 171:3801–13. 10.1111/bph.12749 24780124 PMC4128044

[23] Yuan R Kay A Berg WJ Lebwohl D. Targeting tumorigenesis : development and use of mTOR inhibitors in cancer therapy. J Hematol Oncol. (2009) 2:1–12. 10.1186/1756-8722-2-45 19860903 PMC2775749

[24] Stambolic V Suzuki A Pompa L De Brothers GM Mirtsos C Sasaki T. Negative regulation of PKB / Akt-dependent cell survival by the tumor suppressor PTEN. Cell. (1998) 95:29–39. 10.1016/S0092-8674(00)81780-8 9778245

[25] Easton JB Houghton PJ. mTOR and cancer therapy. Oncogene. (2006) 25:6436–46. 10.1038/sj.onc.1209886 17041628

[26] Land SC Tee AR. Hypoxia-inducible factor 1α is regulated by the mammalian target of Rapamycin (mTOR) *via* an mTOR signaling motif. J Biol Chem. (2007) 282:20534–43. 10.1074/jbc.M611782200 17502379

[27] Capurso G Archibugi L. Molecular pathogenesis and targeted therapy of sporadic pancreatic neuroendocrine tumors. J Hepatobiliary Pancreat Sciepato. (2015) 22:594–601. 10.1002/jhbp.210 25619712

[28] Zhang J Francois R Iyer R Seshadri M Zajac-kaye M Hochwald SN. Current understanding of the molecular biology of pancreatic neuroendocrine tumors. J Natl Cancer Inst. (2013) 105:1005–17. 10.1093/jnci/djt135 23840053 PMC6281020

[29] Shah T Hochhauser D Frow R Quaglia A. Dhillon AP, Caplin ME. Epidermal growth factor receptor expression and activation in neuroendocrine tumours. J Neuroendocr. (2006) 18:355–60. 10.1111/j.1365-2826.2006.01425.x 16629834

[30] Missiaglia E Dalai I Barbi S Beghelli S Falconi M Peruta M. Pancreatic endocrine tumors : expression profiling evidences a role for AKT-mTOR pathway. J Clin Oncol. (2010) 28:245–55. 10.1200/JCO.2008.21.5988 19917848 PMC4288616

[31] Catena L Bajetta E Milione M Ducceschi M Valente M Dominoni F. Mammalian target of rapamycin expression in poorly differentiated endocrine carcinoma: clinical and therapeutic future challenges. Target Oncol. (2011) 6:65–8. 10.1007/s11523-011-0171-z 21468754

[32] Svejda B Kidd M Kazberouk A Lawrence B Pfragner R. Limitations in small intestinal neuroendocrine tumor therapy by mTor kinase inhibition reflect growth factor—mediated PI3K feedback loop activation *via* ERK1 / 2 and AKT. Cancer. (2011) 117:4141–54. 10.1002/cncr.26011 21387274

[33] Jiao Y Shi C Edil BH Wilde RF De Klimstra DS Maitra A. DAXX/ ATRX, MEN1, and mTOR pathway genes are frequently altered in pancreatic neuroendocrine tumors. Science. (2011) 331:1199–204. 10.1126/science.1200609 21252315 PMC3144496

[34] Yuan F Shi M Ji J Shi H Zhou C Yu Y. KRAS and DAXX / ATRX gene mutations are correlated with the clinicopathological features, advanced diseases, and poor prognosis in Chinese patients with pancreatic neuroendocrine tumors. Int J Biol Sci. (2014) 10:957–65. 10.7150/ijbs.9773 25210493 PMC4159686

[35] Öberg K. Genetics and molecular pathology of neuroendocrine gastrointestinal and pancreatic tumors (gastroenteropancreatic neuroendocrine tumors). Curr Opin Endocrinol Diabetes Obes. (2009) 16:72–8. 10.1097/MED.0b013e328320d845 19115524

[36] Asati V Mahapatra DK Bharti SK. European Journal of Medicinal Chemistry PI3K / Akt / mTOR and Ras / Raf / MEK / ERK signaling pathways inhibitors as anticancer agents: structural and pharmacological perspectives. Eur J Med Chem. (2016) 109:314–41. 10.1016/j.ejmech.2016.01.012 26807863

[37] Huang S Houghton PJ Resistance to rapamycin : a novel anticancer drug. Cancer Metastasis. (2001) 20:69–78. 10.1023/A:1013167315885 11831650

[38] Grozinsky-glasberg S Pavel M. Inhibition of mTOR in carcinoid tumors. Target Oncol. (2012) 7:189–95. 10.1007/s11523-012-0225-x 22886906

[39] Duran I Kortmansky J Singh D Hirte H Kocha W Goss G. A phase II clinical and pharmacodynamic study of temsirolimus in advanced neuroendocrine carcinomas. Br J Cancer. (2006) 95:1148–54. 10.1038/sj.bjc.6603419 17031397 PMC2360568

[40] Hobday TJ Qin R Reidy-lagunes D Moore MJ Strosberg J Kaubisch A. Multicenter phase II trial of temsirolimus and bevacizumab in pancreatic neuroendocrine tumors. J Clin Oncol. (2014) 32:1–6. 10.1200/JCO.2014.56.2082 25488966 PMC4417726

[41] Capdevila J Salazar R Halperín I Abad A Yao JC. Innovations therapy : mammalian target of rapamycin (mTOR) inhibitors for the treatment of neuroendocrine tumors. Cancer Metastasis Rev. (2011) 30:27–34. 10.1007/s10555-011-9290-3 21311955

[42] Capozzi M Caterina I De Divitiis C von Arx C Maiolino P Tatangelo F. Everolimus and pancreatic neuroendocrine tumors (PNETs): activity, resistance and how to overcome it. Int J Surg. (2015) 21:S89–94. 10.1016/j.ijsu.2015.06.064 26123382

[43] Tabernero J Rojo F Calvo E Burris H Judson I Hazell K. Dose- and schedule-dependent inhibition of the mammalian target of rapamycin pathway with everolimus : a phase I tumor pharmacodynamic study in patients with advanced solid tumors. J Clin Oncol. (2008) 26:1603–10. 10.1200/JCO.2007.14.5482 18332469

[44] Yao JC Shah MH Ito T Catherine Lombard Bohas EMW Van CE Hobday TJ. Everolimus for advanced pancreatic neuroendocrine tumors. N Engl Lornal Med. (2011) 364:514–23. 10.1056/NEJMoa1009290 21306238 PMC4208619

[45] Yao JC Fazio N Singh S Buzzoni R Carnaghi C Wolin E. Everolimus for the treatment of advanced, non-functional neuroendocrine tumours of the lung or gastrointestinal tract (RADIANT-4): a randomised, placebo-controlled, phase 3 study. Lancet. (2015) 387:1–10. 10.1016/S0140-6736(15)00817-X 26703889 PMC6063317

[46] Verheijen RB Atrafi F Schellens JHM Beijnen JH Huitema ADR Mathijssen RHJ. Pharmacokinetic optimization of everolimus dosing in oncology : a randomized crossover trial. Clin Pharmacokinet. (2017) 57:637–44. 10.1007/s40262-017-0582-9 28762135 PMC5904242

[47] Rinke A Mu H Schade-brittinger C Klose K Barth P Wied M Placebo-controlled, double-blind, prospective, randomized study on the effect of octreotide LAR in the control oftumor growth in patients with metastatic neuroendocrine midgut tumors: a report from the PROMID study group. J Clin Oncol. (2009) 27:4656–63. 10.1200/JCO.2009.22.8510 19704057

[48] Yao JC Phan AT Chang DZ Wolff RA Hess K Gupta S. Efficacy of RAD001 (Everolimus) and octreotide LAR in advanced low- to intermediate-grade neuroendocrine tumors: results of a phase II study. J Clin Oncol. (2008) 26:4311–8. 10.1200/JCO.2008.16.7858 18779618 PMC2653122

[49] Yao JC Catherine Lombard-Bohas EB Kvols LK Rougier P Ruszniewski P Hoosen S. Daily oral everolimus activity in patients with metastatic pancreatic neuroendocrine tumors after failure of cytotoxic chemotherapy : a phase II trial. J Clin Oncol. (2010) 28:69–76. 10.1200/JCO.2009.24.2669 19933912 PMC4295034

[50] Pavel ME Hainsworth JD Baudin E Peeters M Hörsch D Winkler RE. Everolimus plus octreotide long-acting repeatable for the treatment of advanced neuroendocrine tumours associated with carcinoid syndrome (RADIANT-2): a randomised, placebo-controlled, phase 3 study. Lancet. (2011) 378:2005–12. 10.1016/S0140-6736(11)61742-X 22119496

[51] Pavel ME Baudin E Öberg KE Hainsworth JD Voi M Rouyrre N. Efficacy of everolimus plus octreotide LAR in patients with advanced neuroendocrine tumor and carcinoid syndrome : final overall survival from the randomized, placebo-controlled phase 3 RADIANT-2 study. Ann Oncol. (2017) 28:1569–75. 10.1093/annonc/mdx193 28444114 PMC7360141

[52] Bajetta E Catena L Fazio N Pusceddu S Pamela B Blanco G Everolimus in combination with octreotide long-acting repeatable in a first-line setting for patients with neuroendocrine tumors. Cancer. (2014) 15:2457–63. 10.1002/cncr.28726 24752410

[53] Kim HS Shaib WL Zhang C Nagaraju GP. Phase 1b study of pasireotide, everolimus, and selective internal radioembolization therapy for unresectable neuroendocrine tumors with hepatic metastases. Cancer. (2018) 124:1992–2000. 10.1002/cncr.31192 29451701

[54] Kulke MH Ruszniewski P Cutsem EV Lombard-Bohas C Valle JW Herder WWD A randomized, open-label, phase 2 study of everolimus in combination with pasireotide LAR or everolimus alone in advanced, well-differentiated, progressive pancreatic neuroendocrine tumors: COOPERATE-2 trial. Ann Oncol. (2017) 28:1309–15. 10.1093/annonc/mdx078 28327907 PMC7360140

[55] Chan JA Ryan DP Zhu AX Abrams TA Wolpin BM Malinowski P. Phase I study of pasireotide (SOM 230) and everolimus (RAD001) in advanced neuroendocrine tumors. Endocr Relat Cancer. (2012) 19:615–23. 10.1530/ERC-11-0382 22736724 PMC4469068

[56] Chan JA Mayer RJ Jackson N Malinowski P Regan E Kulke MH Phase I study of sorafenib in combination with everolimus (RAD001) in patients with advanced neuroendocrine tumors. Cancer Chemother Pharmacol. (2013) 23:1–7. 10.1007/s00280-013-2118-9 PMC4029418 23475104

[57] Lane HA Wood JM Mcsheehy PMJ Allegrini PR Boulay A Brueggen J. mTOR inhibitor RAD001 (Everolimus) has antiangiogenic/vascular properties distinct from a VEGFR tyrosine kinase inhibitor. Clin Cancer Res. (2009) 15:1612–23. 10.1158/1078-0432.CCR-08-2057 19223496

[58] Hobday TJ Rubin J Holen K Picus J Donehower R Marschke R MC044h, a phase II trial of sorafenib in patients (pts) with metastatic neuroendocrine tumors (NET): a Phase II Consortium (P2C) study. J Clin Oncol. (2007) 25:4504 10.1200/jco.2007.25.18_suppl.4504

[59] Molina AM Feldman DR Voss MH Ginsberg MS Baum MS Brocks DR. Phase 1 trial of Everolimus plus sunitinib in patients with metastatic renal cell carcinoma. Cancer. (2012) 118:1868–76. 10.1002/cncr.26429 21898375 PMC3609026

[60] Wiedmann MW Mössner J. Clinical medicine insights : oncology safety and efficacy of sunitinib in patients with unresectable pancreatic neuroendocrine tumors. Clin Med Insights Oncol. (2012) 6:381–94. 10.4137/CMO.S7350 23226079 PMC3511053

[61] Motzer RJ Barrios CH Kim TM Falcon S Cosgriff T Harker WG. Phase II randomized trial comparing sequential first-line everolimus and second-line sunitinib versus first-line sunitinib and second-line everolimus in patients with metastatic renal cell carcinoma. J Clin Oncol. (2014) 32:2765–72. 10.1200/JCO.2013.54.6911 25049330 PMC5569681

[62] Angelousi A Kamp K Kaltsatou M O'Toole D De Herder W. Sequential everolimus and sunitinib treatment in pancreatic metastatic well-differentiated neuroendocrine tumours resistant to prior treatments. Neuroendocrinology. (2017) 105:394–402. 10.1159/000456035 28122378

[63] Kulke MH Niedzwiecki D Foster NR Fruth B Kunz PL Kennecke HF Randomized phase II study of everolimus (E) vs. everolimus plus bevacizumab (E+B) in patients (Pts) with locally advanced or metastatic pancreatic neuroendocrine tumors (pNET), CALGB 80701 (Alliance). J Clin Oncol. (2015) 33:4005 10.1200/jco.2015.33.15_suppl.4005

[64] Chan JA Blaszkowsky L Stuart K Zhu AX. A prospective, phase 1/2 study of everolimus and temozolomide in patients with advanced pancreatic neuroendocrine tumor. Cancer. (2013) 119:3212–8. 10.1002/cncr.28142 23733618 PMC4308727

[65] Claringbold PG Turner JH. Neuroendocrine tumor therapy with lutetium-177-octreotate and everolimus (NETTLE): a phase i study. Cancer Biother Radiopharm. (2015) 30:261–9. 10.1089/cbr.2015.1876 26181854

[66] Xie J Wang X Proud CG mTOR inhibitors in cancer therapy [version 1; referees : 3 approved] Referee Status. F1000Research. (2016) 5:1–11. 10.12688/f1000research.9207.1 PMC5007757 27635236

[67] Wolin E Mita A Mahipal A Meyer T Bendell J Nemunaitis J A phase 2 study of an oral mTORC1/mTORC2 kinase inhibitor (CC-223) for non-pancreatic neuroendocrine tumors with or without carcinoid symptoms. PLoS ONE. (2019) 14:1–14. 10.1371/journal.pone.0221994 PMC6748410 31527867

[68] Rodrik-Outmezguine VS. Overcoming mTOR resistance mutations with a new-generation mTOR inhibitor. Nature. (2016) 534:272–6. 10.1038/nature17963 27279227 PMC4902179

[69] Fan Q Aksoy O Wong RA Okaniwa M Shokat KM Weiss WA. A kinase inhibitor targeted to mTORC1 drives regression in glioblastoma article a kinase inhibitor targeted to mTORC1 drives regression in glioblastoma. Cancer Cell. (2017) 31:424–35. 10.1016/j.ccell.2017.01.014 28292440 PMC5386178

[70] Moore KN Bauer TM Falchook GS Chowdhury S Patel C Neuwirth R. Phase i study of the investigational oral mTORC1/2 inhibitor sapanisertib (TAK-228): tolerability and food effects of a milled formulation in patients with advanced solid tumours. ESMO Open. (2018) 3:291. 10.1136/esmoopen-2017-000291 29464110 PMC5812400

[71] Chamberlain CE German MS Yang K Wang J Vanbrocklin H Regan M Cancer biology and translational studies a patient-derived xenograft model of pancreatic neuroendocrine tumors identifies sapanisertib as a possible new treatment for everolimus-resistant tumors. Cancer Biol Transl Stud. (2018) 17:1204 10.1158/1535-7163.MCT-17-1204 PMC6279485 30254185

[72] Aksamitiene E Kiyatkin A Kholodenko BN. Cross-talk between mitogenic Ras/MAPK and survival PI3K/Akt pathways: a fine balance. Biochem Soc Trans. (2011) 40:139–46. 10.1042/BST20110609 22260680

[73] Kriegsheim AV Baiocchi D Birtwistle M Sumpton D Bienvenut W Morrice N. Cell fate decisions are specified by the dynamic ERK interactome. Nat Cell Biol. (2009) 11:1458–64. 10.1038/ncb1994 19935650 PMC3839079

[74] Carracedo A. inhibition of mTORC1 leads to MAPK pathway activation through a PI3K-dependent feedback loop in human cancer. J Clin Invest. (2008) 118:3065–74. 10.1172/JCI34739 18725988 PMC2518073

[75] Ruifang M Jianhui M Dechang Z Limin Li HZ. efficacy of combined inhibition of mTOR and ERK/MAPK pathways in treating a tuberous sclerosis complex cell model. J Genet Genomics. (2009) 36:355–61. 10.1016/S1673-8527(08)60124-1 19539245

[76] Wang X Hawk N Yue P Kauh J Ramalingam SS Fu H. Overcoming mTOR inhibition-induced paradoxical activation of survival signaling pathways enhances mTOR inhibitors' anticancer efficacy. Cancer Biol Ther. (2008) 7:1952–8. 10.4161/cbt.7.12.6944 18981735 PMC2762753

[77] Carew JS Kelly KR Nawrocki ST. Mechanisms of mTOR inhibitor resistance in cancer therapy. Target Oncol. (2011) 6:17–27. 10.1007/s11523-011-0167-8 21547705

[78] Zitzmann K Rüden JV Brand S Göke B Lichtl J Spöttl G. Compensatory activation of Akt in response to mTOR and Raf inhibitors—a rationale for dual-targeted therapy approaches in neuroendocrine tumor disease. Cancer Lett. (2010) 295:100–9. 10.1016/j.canlet.2010.02.018 20356670

[79] Fazio N. Neuroendocrine tumors resistant to mammalian target of rapamycin inhibitors: a difficult conversion from biology to the clinic. World J Clin Oncol. (2015) 6:194–8. 10.5306/wjco.v6.i6.194 26677429 PMC4675901

[80] Reilly KEO Rojo F She Q Reilly KEO Rojo F She Q. mTOR inhibition induces upstream receptor tyrosine kinase signaling and activates Akt. Cancer Res. (2006) 66:1500–8. 10.1158/0008-5472.CAN-05-2925 16452206 PMC3193604

[81] Zhang H Griffin JD Kwiatkowski DJ Zhang H Bajraszewski N Wu E. PDGFRs are critical for PI3K/Akt activation and negatively regulated by mTOR. J Clin Invest. (2007) 117:730–8. 10.1172/JCI28984 17290308 PMC1784000

[82] Zhang H Carpenter CL David J Zhang H Cicchetti G Onda H. Loss of Tsc1/Tsc2 activates mTOR and disrupts PI3K-Akt signaling through downregulation of PDGFR. J Clin Invest. (2003) 112:1223–33. 10.1172/JCI200317222 14561707 PMC213485

[83] Elke Tatjana Aristizabal Prada GS Maurer J Lauseker M Koziolek EJ Schrader J Grossman A. The role of GSK3 and its reversal with GSK3 antagonism in everolimus resistance. Endocr Relat Cancer. (2018) 25:893–908. 10.1530/ERC-18-0159 29895527 PMC7439002

[84] Antonuzzo L Del Re M Barucca V Spada F Meoni G Restante G. Critical focus on mechanisms of resistance and toxicity of m-TOR inhibitors in pancreatic neuroendocrine tumors. Cancer Treat Rev. (2017) 57:28–35. 10.1016/j.ctrv.2017.05.001 28535439

[85] Beyens M Vandamme T Peeters M Van Camp G De Beeck KO. Resistance to targeted treatment of gastroenteropancreatic neuroendocrine tumors. Endocrine-Related Cancer. (2019) 26:R109–30. 10.1530/ERC-18-0420 32022503

[86] Karar J Maity A. PI3K/AKT/mTOR pathway in angiogenesis. Front Mol Neurosci. (2011) 4:1–8. 10.3389/fnmol.2011.00051 22144946 PMC3228996

[87] Dormond-meuwly A Roulin D Dufour M Benoit M Demartines N Dormond O. The inhibition of MAPK potentiates the anti-angiogenic efficacy of mTOR inhibitors. Biochem Biophys Res Commun. (2011) 407:714–9. 10.1016/j.bbrc.2011.03.086 21439267

[88] Katsha A Wang L Arras J. Activation of EIF4E by aurora kinase a depicts a novel druggable axis in everolimus-resistant cancer cells. Clin Cancer Res. (2017) 23:3756–68. 10.1158/1078-0432.CCR-16-2141 28073841 PMC5503809

[89] Aguirre D Boya P Bellet D Faivre S Troalen F Benard J. Bcl-2 and CCND1/CDK4 expression levels predict the cellular effects of mTOR inhibitors in human ovarian carcinoma. Apoptosis. (2004) 9:797–805. 10.1023/B:APPT.0000045781.46314.e2 15505422

[90] Modlin IM Kidd M Malczewska A Drozdov I Bodei L Matar S. The NETest: the clinical utility of multigene blood analysis in the diagnosis and management of neuroendocrine tumors. Endocrinol Metab Clin N A. (2018) 47:485–504. 10.1016/j.ecl.2018.05.002 30098712 PMC6716518

[91] Lv Y Han X Zhang C Fang Y Pu N Ji Y. Combined test of serum CgA and NSE improved the power of prognosis predicition of NF-pNETs. Endocr Connect. (2017) 7:1–34. 10.1530/EC-17-0276 29191920 PMC5776672

[92] Baudin E Wolin E Castellano D Kaltsas G Panneerselvam A Tsuchihashi Z Correlation of PFS with early response of chromogranin A and 5-hydroxyindoleacetic acid levels in Pts with advanced neuroendocrine tumours: phase III RADIANT-2 study results. Eur J Cancer. (2011) 47:S460 10.1016/S0959-8049(11)71875-5

[93] Martins D Spada F Lambrescu I Rubino M Cella C Gibelli B. Predictive markers of response to everolimus and sunitinib in neuroendocrine tumors. Target Oncol. (2017) 12:611–22. 10.1007/s11523-017-0506-5 28634872

[94] Zatelli MC Fanciulli G Malandrino P Ramundo V Faggiano A Colao A. Predictive factors of response to mTOR inhibitors in neuroendocrine tumours. Endocr Relat Cancer. (2016) 23:173–83. 10.1530/ERC-15-0413 26666705

[95] Gelsomino F Casadei-Gardini A Caputo F Rossi G Bertolini F Petrachi T. mTOR pathway expression as potential predictive biomarker in patients with advanced neuroendocrine tumors treated with everolimus. Cancers. (2020) 12:1201. 10.3390/cancers12051201 32397669 PMC7281483

[96] Meric-bernstam F Akcakanat A Chen H Do K Sangai T Adkins F. PIK3CA/PTEN mutations and Akt activation as markers of sensitivity to allosteric mTOR inhibitors. Clin Cancer Res. (2012) 18:1777–90. 10.1158/1078-0432.CCR-11-2123 22422409 PMC3307149

[97] Delbaldo C Albert S Dreyer C Sablin M-P Serova M Raymond E. Predictive biomarkers for the activity of mammalian target of rapamycin (mTOR) inhibitors. Target Oncol. (2011) 6:119–24. 10.1007/s11523-011-0177-6 21533544

[98] Nicolantonio FD Biffo S Bardelli A Nicolantonio FD Arena S Tabernero J. Deregulation of the PI3K and KRAS signaling pathways in human cancer cells determines their response to everolimus. J Clin Invest. (2010) 120:2858–66. 10.1172/JCI37539 20664172 PMC2912177

[99] Serra S Zheng L Hassan M Phan AT Woodhouse LJ Yao JC The FGFR4-G388R single-nucleotide polymorphism alters pancreatic neuroendocrine tumor progression and response to mTOR inhibition therapy. AACR J. (2012) 12:5683–92. 10.1158/0008-5472.CAN-12-2102 22986737

[100] Cros J Moati E Raffenne J Hentic O Svrcek M Mestier Ld. Gly388Arg FGFR4 polymorphism is not predictive of everolimus efficacy in well-differentiated digestive neuroendocrine tumors. Neuroendocrinology. (2016) 103:495–9. 10.1159/000440724 26335532

[101] Bellister SA Zhou Y Sceusi E Ellis LM Yao JC Prediction of prognosis in patients treated with everolimus for extrapancreatic neuroendocrine tumors by a single nucleotide polymorphism in PHLPP2. J Clin Oncol. (2013) 31:163 10.1200/jco.2013.31.4_suppl.163

[102] Falletta S Partelli S Rubini C Nann D Doria A Marinoni I. mTOR inhibitors response and mTOR pathway in pancreatic neuroendocrine tumors. Endocr Relat Cancer. (2016) 23:883–91. 10.1530/ERC-16-0329 27697900

[103] Grabiner BC Nardi V Birsoy K Possemato R Shen K Sinha S. A diverse array of cancer-associated MTOR mutations are hyperactivating and can predict rapamycin sensitivity. Cancer Discov. (2014) 4:554–63. 10.1158/2159-8290.CD-13-0929 24631838 PMC4012430

[104] Roviello G Zanotti L Venturini S Bottini A Generali D. Role of targeted agents in neuroendocrine tumours: results from a meta-analysis. Cancer Biol Ther. (2016) 17:883–8. 10.1080/15384047.2016.1210735 27414404 PMC5036406

[105] Kaderli RM Spanjol M Kollár A Bütikofer L Gloy V Dumont RA. Therapeutic options for neuroendocrine tumors a systematic review and network meta-analysis. JAMA Oncol. (2019) 5:480–9. 10.1001/jamaoncol.2018.6720 30763436 PMC6459123

[106] Ravaud A Urva SR Grosch K Cheung WK Anak O Sellami DB. Relationship between everolimus exposure and safety and efficacy: meta-analysis of clinical trials in oncology. Eur J Cancer. (2014) 50:486–95. 10.1016/j.ejca.2013.11.022 24332451

[107] Mujica-Mota R Varley-Campbell J Tikhonova I Cooper C Griffin E Haasova M. Everolimus, lutetium-177 DOTATATE and sunitinib for advanced, unresectable or metastatic neuroendocrine tumours with disease progression: a systematic review and cost-effectiveness analysis. Health Technol Assess. (2018) 22:1–325. 10.3310/hta22490 30209002 PMC6151360

[108] Lee L Ito T Jensen RT. Everolimus in the treatment of neuroendocrine tumors: efficacy, side-effects, resistance, and factors affecting its place in the treatment sequence. Expert Opin Pharmacother. (2018) 19:909–28. 10.1080/14656566.2018.1476492 29757017 PMC6064188

[109] Pan J Bao Q Enders G. The altered metabolic molecular signatures contribute to the RAD001 resistance in gastric neuroendocrine tumor. Front Oncol. (2020) 10:546. 10.3389/fonc.2020.00546 32373532 PMC7186336

